# Usefulness of leadless pacemaker implantation to continue chemotherapy for Burkitt's lymphoma without device infection despite repeated systemic infections

**DOI:** 10.1002/joa3.12837

**Published:** 2023-02-26

**Authors:** Yuichi Nagamatsu, Katsunori Okajima, Tomoyuki Nakanishi, Atsuo Okamura, Yoshio Ohnishi

**Affiliations:** ^1^ Department of Cardiology Kakogawa Central City Hospital Hyogo Japan; ^2^ Department of Medical Oncology/Hematology Kakogawa Central City Hospital Hyogo Japan

**Keywords:** cancer, chemotherapy, infection, leadless pacemaker, malignant lymphoma

Initiating treatment, including chemotherapy, promptly after the diagnosis of malignancy is important; however, chemotherapy frequently induces bradyarrhythmias.^1^ Complications of pacemaker therapy, particularly device infection, should not be a rate‐limiting step in treating malignancy. However, no clear recommendations for device selection exist in the guidelines. Here, we describe a case where a leadless pacemaker was useful in a patient with repeated systemic infections without device infection.

A 62‐year‐old female patient without a previous medical history was admitted for a close examination of anorexia (Day 1). A tumor was found in the ileum of the ascending colon, which was diagnosed as Burkitt's lymphoma (Figure 1A). Echocardiography showed a good left ventricular ejection fraction of 56% before the start of chemotherapy, and the first course of rituximab in combination with fractionated cyclophosphamide, vincristine, doxorubicin, and dexamethasone alternating with rituximab in combination with high‐dose methotrexate and cytarabine (R‐hyper‐CVAD/MA) therapy was administered from Day 12 to Day 32. Tumor lysis syndrome developed in the patient requiring hemodialysis immediately after starting the therapy. On Day 24, the patient was treated with 12 days of antibiotic therapy for *Pseudomonas aeruginosa* bacteremia. On Day 37, a central venous port was placed through the right subclavian vein, and a ureteral catheter was placed for the right ureteral obstruction. On Day 41, the patient experienced a 12‐s pause during sleep because of a paroxysmal atrioventricular block (Figure 2), which required temporary pacing. The implantation of a pacemaker was considered; however, her weight reduced from 53.0 kg to 39.5 kg, her body mass index (BMI) decreased from 22.6 to 16.9 kg/m^2^ with anorexia, and she was undernourished with a serum albumin level of 2.5 g/dL. She also had anemia (Hb 7.5 g/dL) because of hemorrhage caused by cytomegalovirus enteritis. Because of a history of systemic infection, the patient was considered a high‐risk case for device infection with a transvenous pacemaker. In contrast, she was also a high‐risk case for pericardial effusion and cardiac tamponade during leadless pacemaker implantation because of her BMI <20 kg/m^2^, she was female and had non‐atrial fibrillation. After careful discussion in a joint conference among the various departments, leadless pacemaker implantation (Micra™ VR transcatheter pacing system, Medtronic) was performed on Day 47. Although the small right ventricle made the delivery catheter difficult to maneuver, the procedure was completed without complications after one deployment (Figure 3). After resuming chemotherapy from Day 54 as the first course, she experienced pneumocystis pneumonia (Day 92), paralytic ileus (Day 118), methicillin‐resistant coagulase‐negative staphylococci bacteremia (Day 120), sacral pressure ulcers (Day 121), and aspiration pneumonia (Day 141), which resulted in abandonment after three courses. During this period, percent ventricular pacing remained between 0.1% and 0.5%. Although chemotherapy could only be continued for three of the eight courses, the patient's subsequent course was excellent, and the tumor was in nearly complete remission (Figure 1B). Positron emission tomography/computed tomography imaging with [^18^F]‐fluorodeoxyglucose ([^18^F]‐FDG‐PET/CT) performed 6 months after leadless pacemaker implantation showed physiological accumulation in the heart; however, no accumulation was observed on the leadless pacemaker with suspected vegetation (Figure 4). More than 1 year after implantation, the patient continued attending outpatient clinics without developing device infection or lymphoma exacerbation.

**Figure 1:**
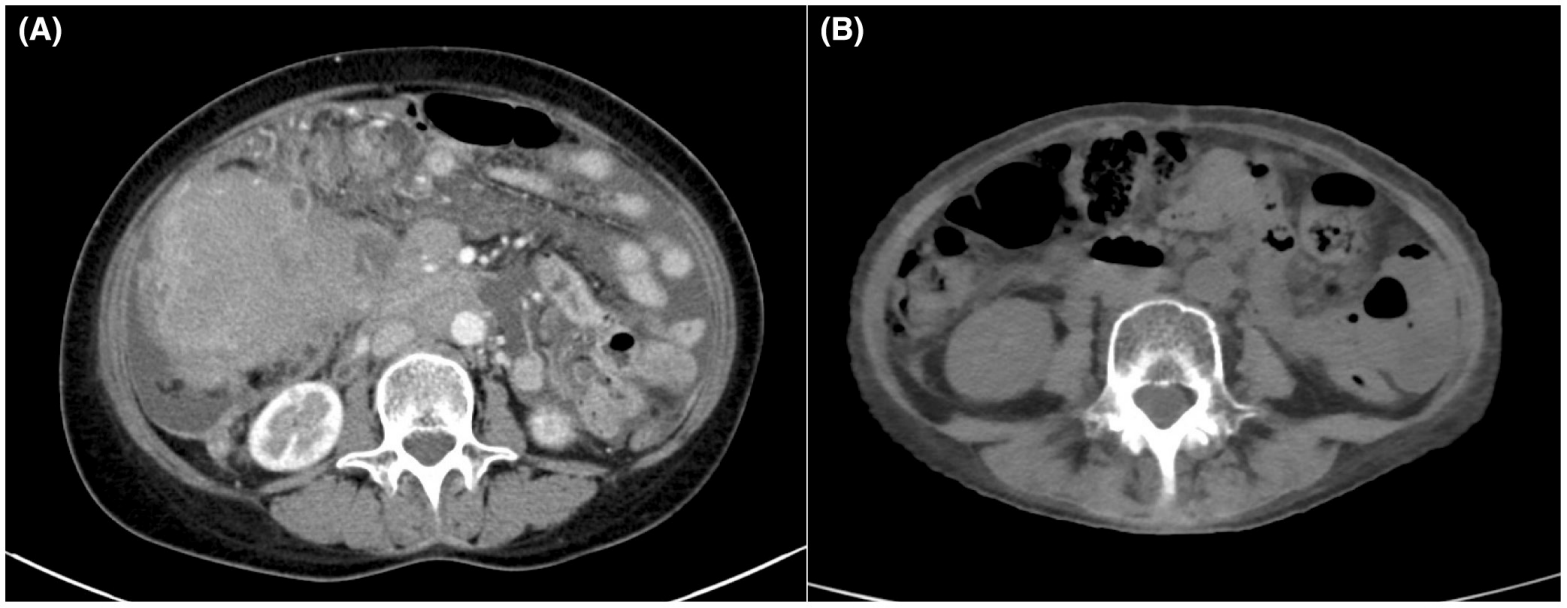
CT images of the abdomen.(A) Arterial phase CT before starting chemotherapy shows a tumor in the ileum and ascending colon. (B) Plane CT performed 7 months after the start of chemotherapy reveals regression of the tumor. CT: computed tomography.

**Figure 2:**
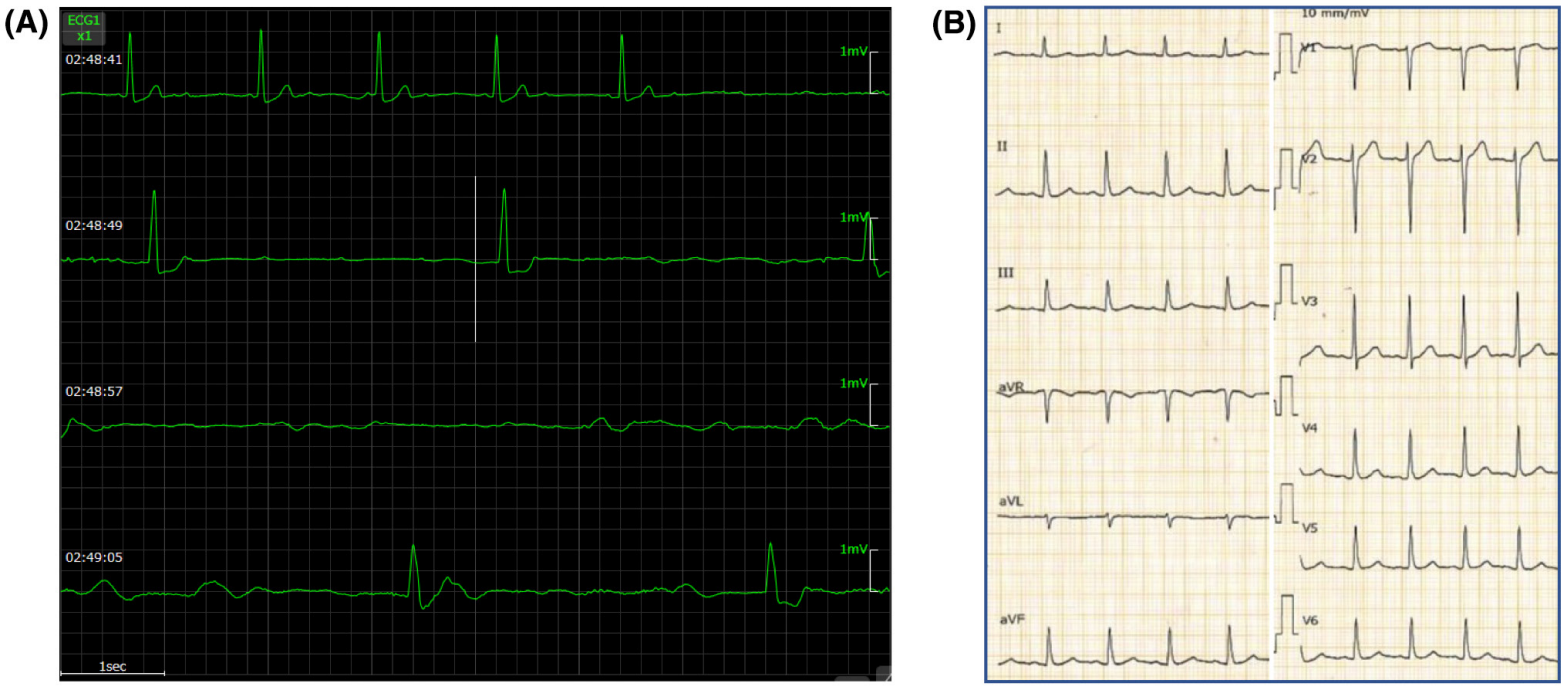
ECG findings. (A) Monitor ECG during sleep detected paroxysmal atrioventricular block. (B) 12‐Lead ECG recorded at a paper speed of 25 mm/s after normalization of the pulse indicates sinus rhythm and no abnormality. ECG, electrocardiogram.

**Figure 3:**
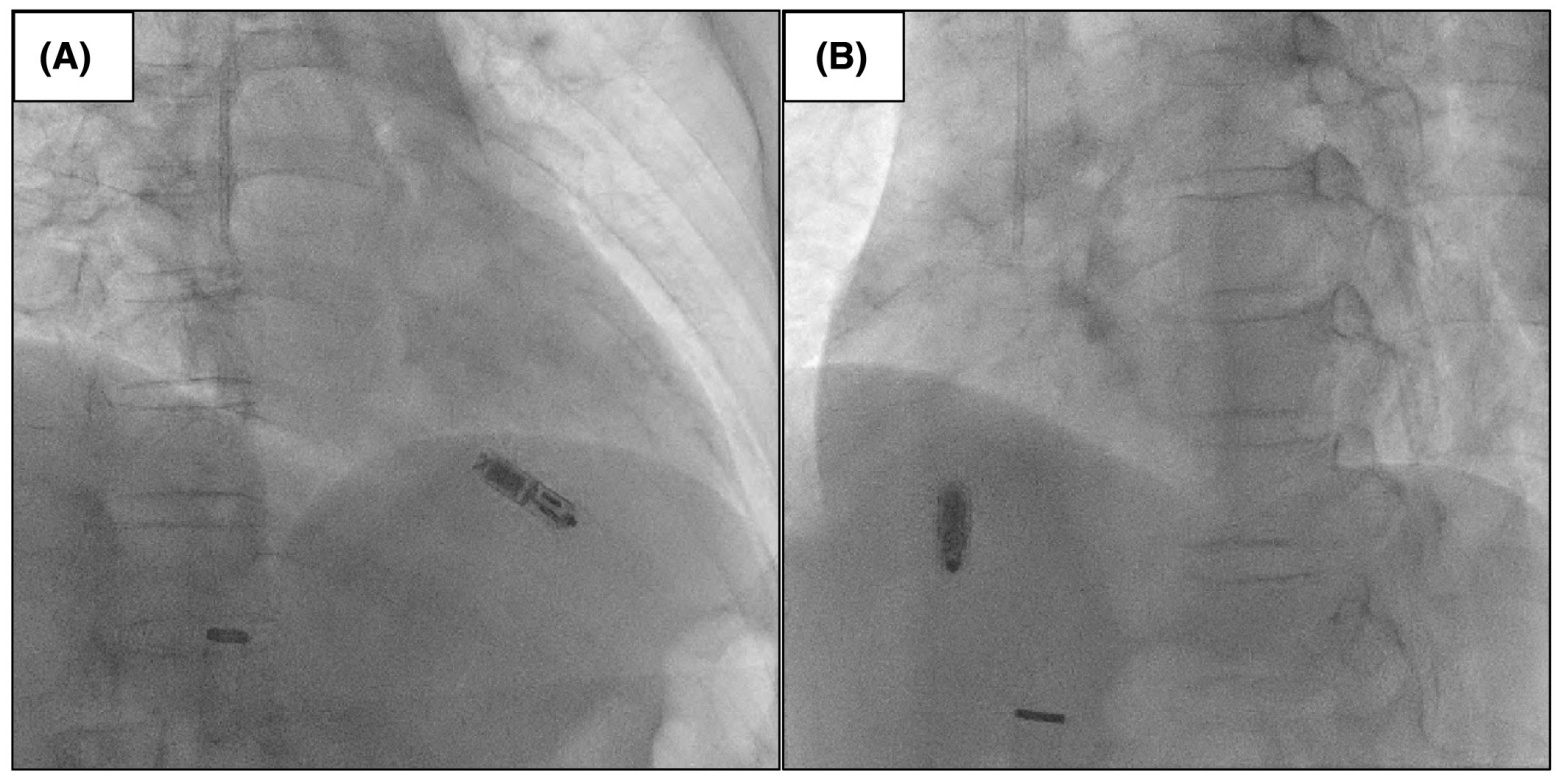
Fluoroscopic images of leadless pacemaker implantation. (A) RAO 30° (B) LAO 45°. RAO, right anterior oblique; LAO, left anterior oblique.

**Figure 4:**
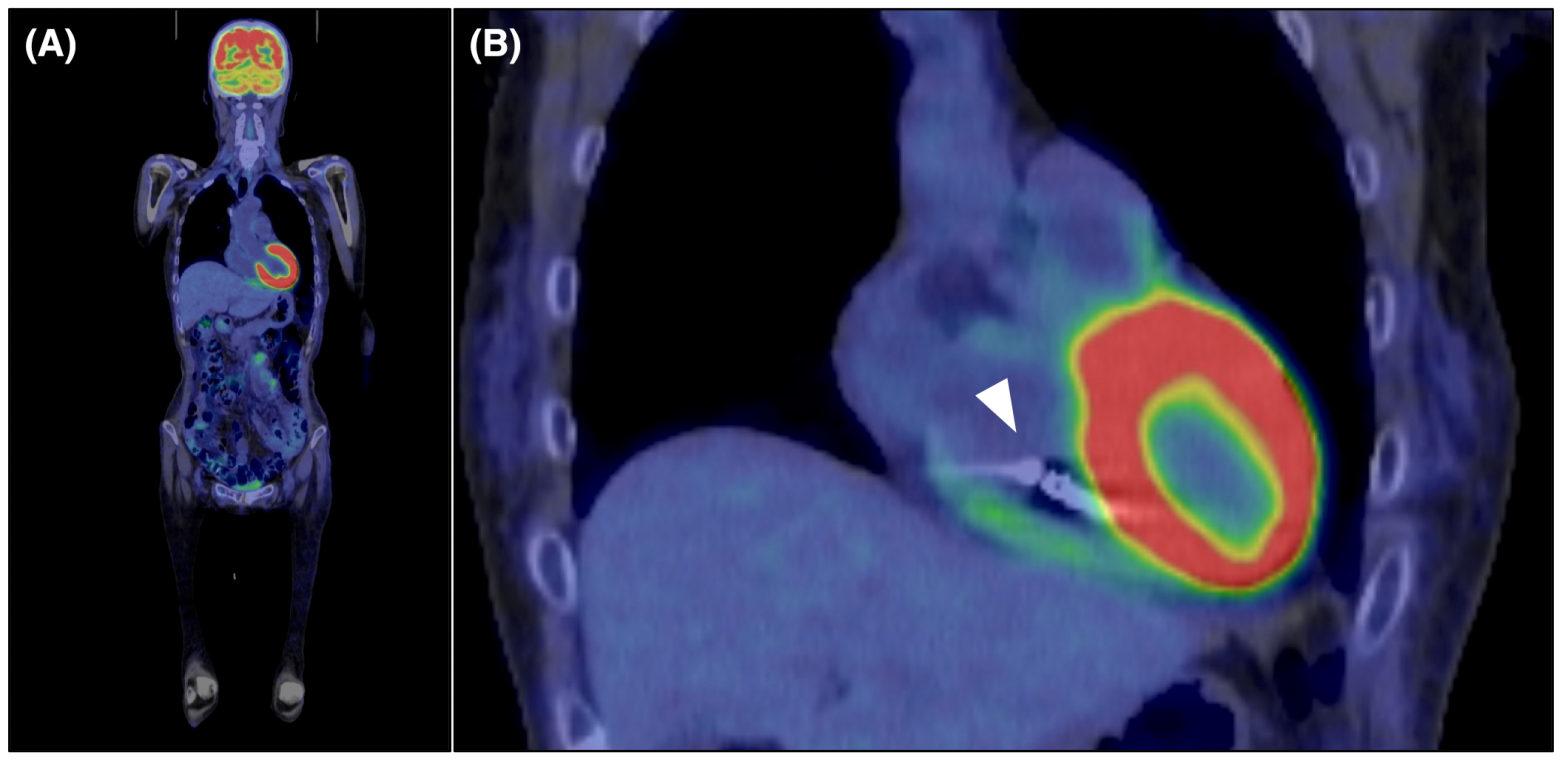
[^18^F]‐FDG‐PET/CT images 7 months after the start of chemotherapy. (A) Overall image shows physiological accumulation in the heart. No abnormal accumulations suspicious of lymphoma exacerbation are observed. (B) A magnified image of the right ventricular apex reveals no abnormal accumulation around the leadless pacemaker body (white arrowhead). [^18^F]‐FDG‐PET/CT, positron emission tomography/computed tomography imaging with [^18^F]‐fluorodeoxyglucose.

Among the drugs used in R‐hyper‐CVAD/MA therapy, rituximab, cyclophosphamide, doxorubicin, methotrexate, and cytarabine can cause sick sinus syndrome and atrioventricular block.^1^ Therefore, there is always a risk of bradyarrhythmia until chemotherapy is completed, which is why a pacemaker is necessary in patients such as in our case. A previous population‐based study reported a 3.7% cumulative incidence of sepsis 1 year after the diagnosis of malignancy and a mortality rate of 35.5% after sepsis.^2^ To our knowledge, the specific probability of pacemaker infection caused by individual chemotherapy regimens, including R‐hyper‐CVAD/MA therapy, has not been reported. However, a meta‐analysis and systematic review of 60 studies reported concomitant malignancy as a risk factor for device infection with an odds ratio of 2.23. It is assumed that the risk of device infection increased because of immunosuppression associated with chemotherapy and the deterioration of the general condition following malignancy.

Leadless pacemakers, which have spread rapidly recently, are resistant to infection because of the absence of a pocket and leads, insufficient direct surgeon contact with the device, small surface area, early encapsulation, antimicrobial properties of the parylene coating, and the fact that they are implanted in the right ventricle with fast blood flow.^3^ The European Society of Cardiology guidelines recommend that leadless pacemakers should be considered as an alternative to transvenous pacemakers in patients who are at particularly high risk of device pocket infection, such as patients with a history of infection or those on hemodialysis.^1^ Of the 1820 patients enrolled in the Micra postapproval registry,^4^ 105 had a leadless pacemaker implanted within 30 days of infected device removal. Of these, 37% had a leadless pacemaker implanted on the same day as the removal of the infected device, and no reports were noted of patients requiring removal of the leadless pacemaker because of infection at follow‐up. However, there have been some reported cases of leadless pacemakers requiring removal because of vegetation adhering to the devices.^5^ Because patients indicated for leadless pacemakers are at a high risk of infection, strict systemic management is required after device implantation.

We demonstrated a case where leadless pacemaker implantation was effective in allowing continued chemotherapy for Burkitt's lymphoma without device infection, even after repeated systemic infections. Immunocompromised status because of malignancy and its treatment are risk factors for device infections. The selection of leadless versus transvenous pacemakers in patients with cancer is currently determined by the operator without guideline recommendations. Therefore, a leadless pacemaker with high resistance to infection may be a useful treatment option for patients with cancer. It is important to emphasize that appropriate chemotherapy should not be delayed because of the risk of device infection.

## CONFLICT OF INTEREST STATEMENT

Authors declare no conflict of interests for this article

## PATIENT CONSENT STATEMENT

The authors obtained consent from the patient

## CLINICAL TRIAL REGISTRATION

N/A

## Data Availability

Available upon reasonable request.
